# COVID-19 Pandemic Lockdown: An Excellent Opportunity to Study the Effects of Trawling Disturbance on Macrobenthic Fauna in the Shallow Waters of the Gulf of Gabès (Tunisia, Central Mediterranean Sea)

**DOI:** 10.3390/ijerph19031282

**Published:** 2022-01-24

**Authors:** Nawfel Mosbahi, Jean-Philippe Pezy, Jean-Claude Dauvin, Lassad Neifar

**Affiliations:** 1 Laboratoire de Biodiversité Marine et Environnement, Faculté des Sciences de Sfax, Université de Sfax, BP 1171, Sfax 3038, Tunisia; lassad.naifar@fss.rnu.tn; 2 Laboratoire Morphodynamique Continentale et Côtière, Normandie University, UNICAEN, CNRS, UMR 6143 M2C, 24 Rue des Tilleuls, 14000 Caen, France; jean-philippe.pezy@unicaen.fr (J.-P.P.); jean-claude.dauvin@unicaen.fr (J.-C.D.)

**Keywords:** bottom trawling, COVID-19 pandemic lockdown, environmental impacts, macrobenthic fauna, tidal channels, central Mediterranean Sea

## Abstract

This study describes for the first time in the central Mediterranean Sea the effects of bottom trawling on macrobenthic fauna in tidal channels of the Kneiss Islands in the Gulf of Gabès, Tunisia. Following a BACI protocol, two control stations (protected by artificial reefs) and two trawled stations (impacted stations) were sampled during a period with the absence of bottom trawling activity (the COVID-19 pandemic lockdown period from March to May 2020) and during a trawled period. Although bottom trawling had no impact on sediment composition, this anthropogenic activity reduced the concentration of dissolved oxygen and had a noticeable effect on water column turbidity. The absence of trawling led to a significant increase in biomass, number of species, and abundance of total macrofauna. This illustrated the negative effect of trawling activity in shallow waters and the high resilience of macrobenthic communities of the tidal ecosystem of the Kneiss Islands. In the future, it would be very important to control the use of this destructive fishing gear due to its negative impact on the marine habitat and macrofauna, which represents essential prey for fishes and birds living in this protected area.

## 1. Introduction

Bottom trawling is one of the most harmful anthropogenic activities on both shallow and deep marine ecosystems [1,2,3]. It is a relatively non-selective fishing method with global negative impacts on benthic communities and habitats [4,5,6]. Bottom trawling provokes sediment re-suspension; decreases macrofaunal bioturbation processes; and removes, injures, or kills a wide range of sedentary organisms. It also induces changes in the population demography and can have dire consequences on ecosystem structures and functions [7,8,9,10].

The Gulf of Gabès, located in the central part of the Mediterranean, covers the second-widest continental shelf area (35,900 km^2^) and is characterized by unique geomorphological, climatic, and oceanographic conditions. This gulf has an extensive network of tidal channels and very gentle slopes [11]. The tidal channel environments are of major ecological importance, being considered among the main pathways of passage and migration for several commercial marine species (fish and shrimp) and providing important habitats for the juveniles of many inshore fish species [12,13]. Therefore, the Gulf of Gabès is an important nursery for several fish species and represents one of the main target areas for fishing activities in Tunisia; moreover, it is among the most highly productive zones in the Mediterranean Sea [14]. The favourable geomorphologic and climatic conditions are combined to support one of the most productive ecosystems around the small tidal channels in the Gulf of Gabès [14,15]. These tidal features are very attractive for many kinds of fishing activity, especially bottom trawling [16]. Around the Kneiss Islands, tidal channels are visible only at low tide; this unique system in the Mediterranean Sea represents the highest energy environment of the Gulf of Gabès and is sensitive to anthropogenic activities and climate change [17,18]. These conditions favour the circulation of seawater, sediments, organic matter, and nutrients between terrestrial and coastal marine environments [19]. Due to their diversity of birds, the Kneiss Islands have been designated as a ‘Specially Protected Area of Mediterranean Importance’ (SPAMI) in 2001, an ‘Important Bird Area’ (IBA) in 2003, and as a ‘RAMSAR site’ since 2007. Nevertheless, despite international protection, intensive human activities occur in this Marine Protected Area [20]. In the tidal channels of the Kneiss Islands, fishers use traditional and impacting fishing gear such as the small bottom trawl known locally as “Kiss”. The Kiss trawlers usually display all of the characteristics and gears that can be found on regular trawlers: a net with a lead line; a float line; and a cod end, tied on both sides to otter doors that are usually wooden on Kiss trawlers. The main differences between a Kiss trawler and a regular one are the size of the vessel and those of the net. The mesh size of the Kiss nets is much smaller (18 versus more than 28 mm) than on a regular trawler, which makes them less selective and liable to producing large amounts of by-catch. Kiss trawlers are small versions of the regular ones; they rarely reach 10 m in length, are mainly manufactured with wood, and are equipped with a power winch and an iron bar on the stern of the boat to tow the net. Illegal vessels could operate in areas of shallow water (5 m depth or less), destroying sensitive habitats and spawning grounds, by tearing out the seagrass meadows caused by bottom scraping metal panels towed by the trawler [21].

Such gear unfavourably affects the juveniles of fish and shrimps as well as sensitive benthic habitats such as *Posidonia oceanica* meadows [7,22]. In October 2018, a team of inspectors counted the vessels equipped with unauthorized bottom trawls to estimate the total number of illegal trawlers operating over the whole Gulf of Gabès. Between 400 and 500 of such trawlers were identified in the main ports around the Gulf of Gabès [21]. It is very difficult to recognize and separate illegal Kiss trawlers from other trawlers, as most of them do not display their identification numbers or VMS (vessel monitoring system) used to monitor the location and the activities of commercial fishing vessels required under Tunisian legislation, making legal pursuit and law enforcement harder. We estimated that, around the Kneiss Islands, in the central part of the Gulf of Gabès, approximately 100 vessels carry out trawling activities every day in tidal channels. These vessels came from the ports of Khawala, Skhira, and Mahres. They were small vessels (7 to 10 m of length) equipped with mini trawls to catch target species such as the shrimp *Metapenaeus monoceros* (Fabricius, 1798), several fish species, including *Sparus aurata* Linnaeus, 1758; *Diplodus* spp.; *Solea* spp.; cephalopods *Sepia officinalis* Linnaeus, 1758; and recently, the exotic invasive blue crab *Portunis segnis* (Forskål, 1775).

To study anthropogenic pressures and to assess their effects, ecologists and stakeholders have stressed the key role of the macrofauna in establishing the ecological quality status of benthic communities [23,24] and in assessing the biological integrity of marine systems [25,26]. In fact, benthic invertebrates are considered potentially powerful indicators of marine ecosystem health [24]. Due to their life at the sediment–water interface and their short life spans, these organisms can record both accidental and chronic perturbations in marine environments [27,28,29].

Coronavirus Disease 2019 (COVID-19) is the official name of a respiratory infectious disease caused by a new coronavirus that was first reported in Wuhan, China, and then spread unexpectedly fast across the world on 11 March 2020, when the World Health Organization (WHO) stated the COVID-19 outbreak as a “pandemic public health menace” [30,31] due to its worldwide spread. Since February 2020, affected countries have locked down their cities and industries to restrict the movement of their citizens and to minimize the spread of the virus [30,32]. Despite the negative aspects of coronavirus on human health, this crisis had a positive impact on the natural environment: improvements in air and water quality, and reductions in industrial effluents and in the discharge of other wastes [33,34]. There had also been a decline in the exploitation of natural resources and a strong rise in the successful recruitment of several marine species observed in certain perturbed ecosystems (harbours, industrial coastal zones, etc.), which was attributed to lowered anthropogenic pressures [35,36]. These might be short-term improvements, but they highlighted the severity of anthropogenic impacts worldwide.

In the Gulf of Gabès, a major part of this scientific work was carried out to assess the interaction between fishing with bottom trawling, and vulnerable and protected animals such as sharks, rays, and marine turtles. Data on the impacts of bottom trawling on macrobenthic fauna in the Gulf of Gabès are few and even rare, because a reference period is required to study anthropogenic activities to understand their environmental effects. Many human activities have developed around the Kneiss Islands, including bait digging [20] and clam harvesting [37], and several research protocols have been implemented to study the effects of each activity (see [20,37]).

This work highlights the response of macrobenthic communities when faced with the cessation of destructive fishing activities during the COVID-19 pandemic lockdown period. Therefore, this study was carried out using a BACI (Before After Control Impact) approach, exploiting the COVID-19 pandemic lockdown period to evaluate the impacts of bottom trawling on soft-bottom macrobenthic communities of the tidal channels of the Kneiss Islands in order to set up an effective management plan for artisanal fishing activity in this subtidal ecosystem of the central Mediterranean Sea.

## 2. Materials and Methods

### 2.1. Study Area

Located in the northwestern part of the Gulf of Gabès, between latitudes 34°10′ N and 34°30′ N and between longitudes 10°E and 10°30′ E, the Kneiss islands are characterized by the highest tidal range in the western Mediterranean Sea; the tide is semi-diurnal with an amplitude varying from 0.8 to 2.3 m [11]. Tidal currents have created shallow channels in the Gulf of Gabès that are extensively developed around the Kneiss islands. In these ecosystems, sediments are mainly composed of sand and coarse sand; the sedimentary filling of the tidal channels shows decreasing grain size from downstream to upstream, indicating that the action of tidal currents is stronger during a flood tide than during an ebb tide [19]. Moreover, sediments in the shallowest waters are mainly characterized by sand, whilst gravel is found at intermediate-depth stations and deeper stations are dominated by fine sediment including silt [16]. Intensive human activities take place in this protected area of the Kneiss Islands [16,20,37].

### 2.2. Sampling and Laboratory Procedures

#### 2.2.1. Macrobenthic Sampling and Measurement of Physicochemical Variables

**Figure 1:**
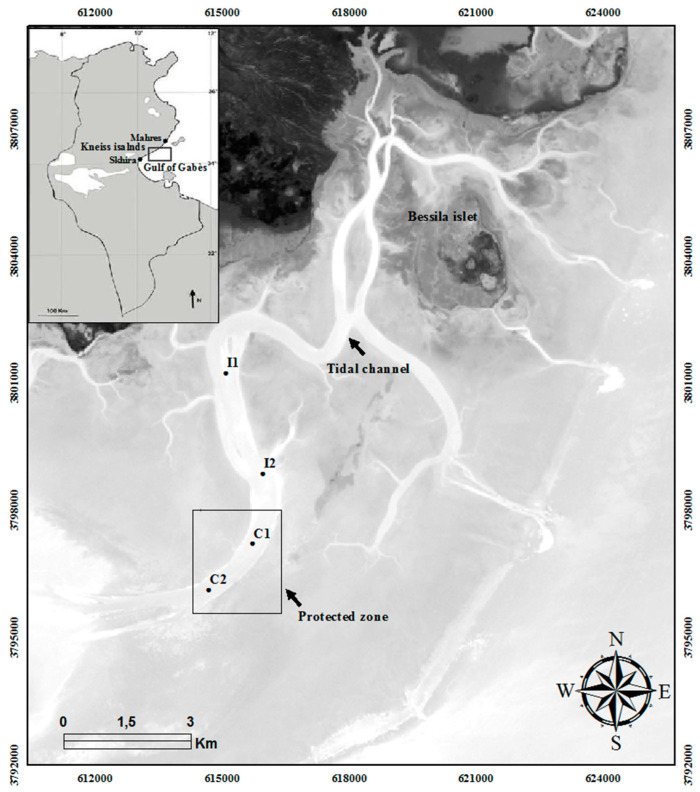
Map of the study area showing the locations of the sampling stations (I1 and I2: trawled zones; C1 and C2: control stations).

**Figure 2:**
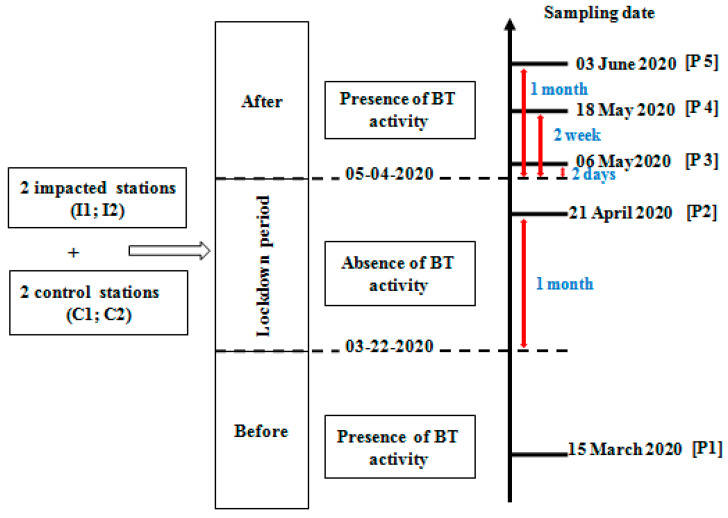
Representation of the sampling strategy used in this study during the five sampling periods, with P1: before lockdown; P2: during lockdown; P3: after two days; P4: after two weeks; P5: one month after the lockdown; and BT: bottom trawling.

Benthic macrofauna sampling was performed between March and June 2020, and carried out in the tidal channels of the Kneiss Islands at two trawled stations (i.e., stations I1 and I2) in zones where the fishers used bottom trawling and at two other control stations (stations C1 and C2: located in a protected zone preserved by artificial reefs since 2014) (Figure 1 and Figure 2). Five sampling campaigns were organized according to a BACI strategy: one during March before the lockdown period, another at the end of the lockdown period (total cessation of trawler fishing activity controlled by the coast guard and the national navy), and three after the lockdown period (Figure 2). Benthic macrofauna sampling was carried out using a Van Veen grab covering an area of about 0.05 m^2^, which penetrated approximately 0.1 m into the sediment. The station positions were accurately determined using a GPS (Global Positioning System, WGS84). For each sampling campaign, five replicates were carried out at each station: four for biological analysis covering a total surface-area of 0.2 m^2^ and one for sediment analysis. Each biological sample was sieved through a 1 mm mesh, fixed with buffered 10% formaldehyde, and stained with Rose Bengal to facilitate sorting. In the laboratory, prior to identification, the samples were washed and the organisms were hand-sorted into major taxonomic groups, identified to the lowest practical taxonomic level (usually species level), and then counted. Biomass was obtained as ash-free dry weight (g AFDW) after drying (60 °C for 48 h) and calcination (500 °C for 5 h). Species names were checked using the World Register of Marine Species list (http://www.marinespecies.org accessed on 20 November 2021). In addition, several environmental factors were measured in situ such as depth (m), turbidity (measured by Suspended Solids Concentration “SSC”; mg L^−1^), temperature (T °C), salinity (measured in situ by WTW multi-meter) and dissolved oxygen concentration (by WTW oximeter; mg L^−1^).

#### 2.2.2. Sediment Grain-Size Analysis and Organic Matter Content

The sediment from each sampling period was homogenized and wet-sieved through a 63 μm mesh to separate muddy (including silt and clay), gravely, and sandy fractions (retained on the sieve). After being oven-dried to constant weight at 60 °C, sediment fractions were separated using a mechanical shaker (column of five sieves with mesh sizes of 1000, 500, 250, 125, and 63 μm) for 10 min. All fractions (including <63 μm) were then weighed to determine their percentage relative abundance. For the organic matter content analyses, sediment samples were dried at 60 °C to constant weight and ground to a fine powder. Organic matter content was determined on the powder samples by ‘loss on ignition’ at 450 °C for 4 h.

### 2.3. Statistical Analyses

Univariate and multivariate analyses were used to evaluate the changes in the structure and composition of macrobenthic assemblages due to bottom trawling. We considered the number of taxa, abundance, and biomass per station. These biological data were used to calculate, at each station, the abundance (ind. m^−2^) and the most common biodiversity indices, i.e., taxonomic richness (number of taxa per 0.2 m²), biomass (g AFDW m^−2^), Shannon index (H’) [38], and Pielou’s evenness (J’) [39].

A Log (x + 1) transformation was applied to the abundance matrix (data for each station were pooled prior to undertaking further analyses) to minimize the influence of the most dominant taxa, before calculating the Bray–Curtis similarities using the statistical package PRIMER [40]. A dendrogram was created with group averages expressed in the cluster mode. Then, a non-parametric multi-dimensional scaling (n-MDS) ordination was applied to the abundance matrix using the Bray–Curtis similarity measure, with the objective of examining the structure of the macrobenthic assemblages. The SIMilarity PERcentages (SIMPER) routine was applied to establish which species contributed most to the observed differences in the data. Analyses of the data were performed using PRIMER^®^ version 6 (Plymouth Routines in Multivariate Ecological Research) package [40]. Permutational multivariate analysis of variance (PERMANOVA) was also conducted to test the significant differences in macrobenthic fauna composition in response to bottom trawling (one-way analysis) [41].

For the biological parameters, a Shapiro–Wilk normality test and a Bartlett’s test for homogeneity of variance were performed prior to each ANOVA to check whether the assumptions of ANOVA were met and if data transformation was necessary. Then, three-way ANOVAs were used to assess the spatiotemporal effect of bottom trawling on taxonomic, abundance, and biomass of macrofauna benthic between sample stations and during each sampling campaign (before lockdown; during lockdown; and after two days, two weeks, and one month of deconfinement). A Tukey Honestly Significant Difference test was employed to determine differences before and after trawling period as well as between the different sediment types. These statistical procedures were performed using the R software vegan package.

## 3. Results

### 3.1. Environmental Variables

There were only a very small proportion of fine particles (<63 μm) in the sediment, representing between 2 and 3.4% at stations I2, C1, and C2 and reaching 16% at I1. The sediment was dominated by coarse sand (500–2000 μm) (68 to 82%), except at I1, which was dominated by medium sand (74%). The organic matter content varied between 2.6% (C2) and 4.1% (I1) (Table 1). No significant statistical changes could be identified in sediment type and organic matter content between sampling stations and between dates (in both cases; *p* > 0.05), showing that the sediment variables were stable throughout the BACI study period from March to June 2020.

**Figure 3:**
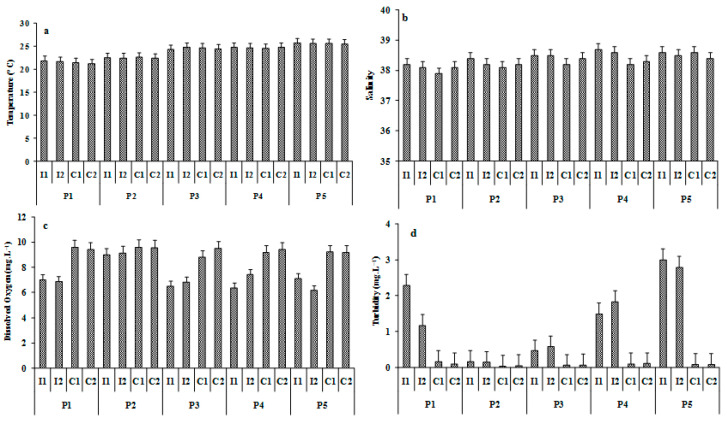
Physicochemical parameters (± SD) during the five sampling periods, with P1: 15 March; P2: 21 April; P3: 6 May; P4: 18 May; and P5: 3 June 2020, were (**a**) temperature (°C), (**b**) salinity, (**c**) dissolved oxygen (mg L^−^^1^), and (**d**) turbidity (Suspended solid concentrations; mg L^−^^1^).

Figure 3 showed the variation in physicochemical variables measured during the sampling period. The dissolved oxygen and the turbidity were significantly different (ANOVA; F = 1.38; *p* < 0.01; F = 2.12; *p* < 0.01, respectively) between the control and impacted stations during the five trawling periods (P1, P3, P4, and P5; illustrated in Figure 2), with higher turbidity values in the trawling stations (I1 and I2) and higher dissolved oxygen values at the control stations (C1 and C2). The impacted stations exhibited lower values of dissolved oxygen and higher values of SSC compared with the control stations during the trawling period and particularly at the last date of sampling. Conversely, no significant differences were identified for temperature and salinity between the stations during the five sampling periods for the both environmental variables (*p* < 0.05) (Table 1).

### 3.2. General Composition of the Benthic Macrofauna

In total, 4846 macrofaunal individuals were identified belonging to 72 taxa and 6 zoological groups unequally distributed among the sampling stations. Annelid polychaetes were dominant (40% of the total number of taxa), followed by crustaceans (34%), mainly amphipods, decapods, and isopods, along with molluscs (21%), mainly bivalves and gastropods. The other three phyla (echinoderms, cnidarians, and tunicates) accounted for only 5% of the total number of taxa. Similarly, the polychaetes dominate numerically (42% of the total abundance) with the species *Cirratulus cirratus* (O. F. Müller, 1776)*, Euclymene lumbricoides* (Quatrefages, 1866)*,* *Amphitritides gracilis* (Grube, 1860), and *Nereis* spp. showing the highest number of individuals.

### 3.3. Impact of Bottom Trawling on the Macrofauna

**Figure 4:**
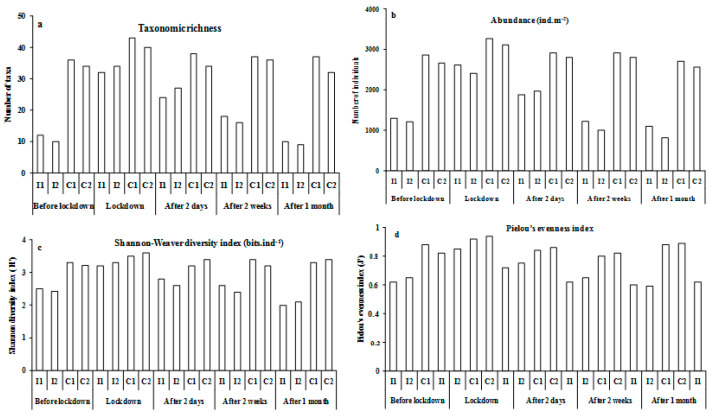
Benthic community parameters: taxonomic richness (**a**), abundance (**b**), Shannon–Wiener index (**c**), and Pielou’s evenness (**d**) with standard deviation for all stations sampled before lockdown; during lockdown; and after two days, two weeks, and one month after confinement.

Before the lockdown period (15 March 2020), the number of taxa and abundances in the four sampled stations were significantly different, with higher values recorded in the control stations than in the impacted stations (F = 3.9; *p* < 0.01; F = 2.1; *p* < 0.01, respectively). During the lockdown period (21 April 2020), when there were no trawling activities, the number of taxa and abundance increased at both impacted stations. After lockdown, abundances and numbers of taxa at both control stations were higher after two days, two weeks, and one month of the period after confinement compared with the impacted stations (Figure 4; Table 2).

H’ and J’ varied significantly between stations (F = 2.18; *p* < 0.01; F = 3.11; *p* < 0.01, respectively) (Table 3), with higher values of these indices corresponding to control stations during the lockdown period (H’= 3.6 bits.ind^−1^; J’= 0.95 in C2) (Figure 4).

**Figure 5:**
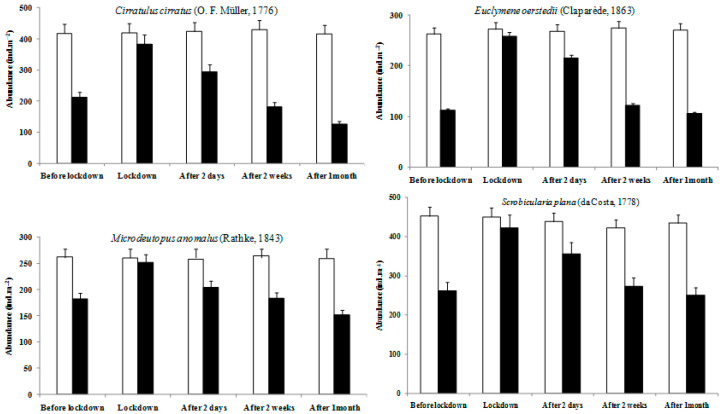
Mean abundance (± SD; *n* = 2) for some representative species at control and impact stations during the five sampling periods (before lockdown; during lockdown; and after two days, two weeks, and one month of deconfinement). White column: control stations; black column: impacted stations.

During the lockdown period, the four stations were dominated by polychaetes (~40%), followed by crustaceans and molluscs. During the trawling period, the faunal composition varied between the control and the impacted stations, with lower values in the impacted stations. The abundance of dominant species such as the infaunal polychaetes *Cirratulus cirratus* (O.F. Müller, 1776)*, Euclymene oerstedii* (Claparède, 1863), the amphipod *Microdeutopus anomalus* (Rathke, 1843), and the bivalve *Scrobicularia plana* (da Costa, 1778) decreased during the trawling periods at trawled sites compared with the control stations. In contrast, the abundances of the latest species were higher during the lockdown period and even two days after the end of the lockdown in all sampled stations (Figure 5).

Several sedentary organisms such as the porifera; echinodermata; cnidaria; and the tubicolous polychaeta (including the Sabellidae *Sabella pavonina* Roule, 1896 and *Branchiomma bombyx* (Dalyell, 1853); the Terebellidae *Terebella lapidaria* Linnaeus, 1767 and *Amphitrite rubra* (Risso, 1826); and the Serpulidae *Serpula vermicularis* Linnaeus, 1767 and *Hydroides dianthus* (Verrill, 1873)) only appeared at trawled stations during the lockdown period and after two days at the end of the lockdown, and then disappeared after two weeks later (18 May 2020). The ANOVA test showed that there were significant difference between biomass recorded during the five sampling periods at all stations (ANOVA; df = 4; F = 21.4; *p <* 0.05) (Table 3). The control stations showed higher biomass compared with both trawled stations during the five sampling campaigns (ANOVA; df = 1; F = 12.2; *p <* 0.01). The biomass of macrobenthic communities was higher at the control stations during the four fishing periods compared with the trawled stations (I1 and I2). During the lockdown period (P2), the biomass increased at the impacted stations simultaneously with the increase in abundance. Equally, the biomass of the main zoological group showed significant variation during the five sampling periods (ANOVA; df = 4; F = 112.4; *p <* 0.05). The biomass of polychaetes recorded at trawled stations gradually decreased with the increase in the number of trawling days (see Table 2).

**Figure 6:**
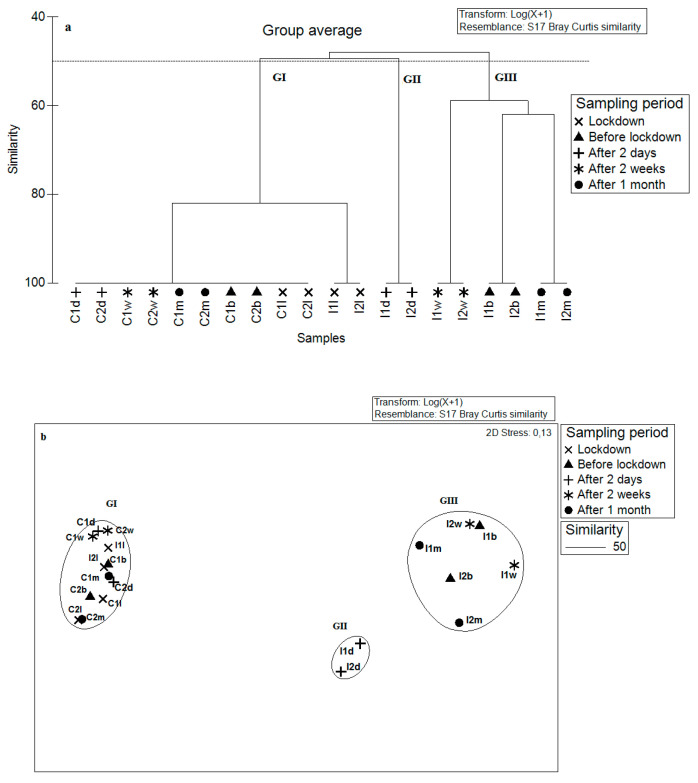
Dendrogram (**a**) and n-MDS ordination plot (**b**) of Bray–Curtis similarities from abundance data (Log (x + 1) transformation) for impacted (I1 and I2) and control (C1 and C2) sampling before lockdown (b); during lockdown (l); and after two days (d), two weeks (w), and one month (m) of deconfinement. Three groups of stations are identified by cluster analysis at a 50% similarity level.

The dendrogram and n-MDS (Figure 6) showed a remarkable spatial separation between the sampled stations in all three groups. The first group (GI) corresponded to both control stations sampled during the five campaigns plus both impacted stations sampled during the lockdown period. The second group (GII) corresponded to the two impacted stations sampled after only two days of deconfinement (resumption of bottom trawling activity) (I1d and I2d). The last group (GIII) gathered the impacted stations sampled before lockdown, after two weeks and after one month of deconfinement.

SIMPER was used here to illustrate the biological reasons for the clustering of stations with (before and after lockdown) and without bottom trawling activity (during lockdown period) (Table 4) by showing the group similarity identifying species contributing most to the similarity between groups. The first group (GI; 84.54% contribution to total similarity) was strongly represented by the polychaetes *Cirratulus cirratus, Euclymene oerstedii, Hediste diversicolor, Amphitritides gracilis,* and *Euclymene lumbricoides* and the amphipods *Monocorophium acherusicum* and *Microdeutopus anomalus.* The second group (GII; 59.43%) was represented by the polychaetes *Cirratulus cirratus, Hilbigneris gracilis*, and *Perinereis cultrifera;* the molluscs *Cerithium scabridum, Scrobicularia plana,* and *Tricolia speciosa*; and the amphipods *Monocorophium insidiosum* and *Dexamine spinosa.* The last group (GIII; 27.82%) corresponded to the impacted stations sampled before lockdown and after two weeks and then one month of deconfinement. This group was represented by the amphipods *Microdeutopus gryllotalpa, Cymadusa filosa*, and *Dexamine spinosa* and the molluscs *Gibbula ardens, Loripes orbiculatus*, and *Calliostoma zizyphinum* (Table 4). The PERMANOVA analysis showed that the three groups are statistically highly separated (PERMANOVA; F = 23.2; *p* < 0.01).

## 4. Discussion

Bottom trawling has global negative impacts on marine biodiversity and habitats and is already considered among the anthropogenic pressures that threaten marine environments [5,6,8,10,42]. This destructive fishing activity is very widespread in the Mediterranean and practiced intensively in the Gulf of Gabès region. In the Mediterranean Sea, many studies have demonstrated the negative environmental impacts of bottom trawling on *Posidonia oceanica* meadows, marine fish productivity, and macroinvertebrate fauna diversity [43,44,45]. According to different observations in several Mediterranean fisheries, trawling activities have been responsible for the bulk of discards and the decline in fish stocks during the last few decades in the Mediterranean region [5,44].

To study the impacts of bottom trawling on macrobenthic communities in tidal channels of the Kneiss Islands, we use a BACI approach applied before and after the COVID-19 pandemic lockdown in the Gulf of Gabès. In this study, we evaluate for the first time the effects of trawling on the benthic fauna of soft bottom substrates. Many recent studies have indicated that the COVID-19 lockdown had important positive impacts in reducing several human activities and improving environmental quality [35,36]. Similarly, the before/after COVID-19 pandemic lockdown approach is an excellent opportunity used to assess the impacts of trawling on benthic communities in the central part of the Mediterranean Sea.

The macrofauna of the tidal channels of Kneiss Islands is mainly composed of polychaetes belonging to the families *Cirratulidae, Maldanidae, Nereididae*, and *Lumbrineridae*, followed by the amphipods represented by the *Gammaridae, Dexaminidae, Corophiidae*, and *Aoridae*. During the five sampling periods (Figure 2), the control stations showed higher diversity indices (taxonomic richness, abundance, and biomass) compared with the trawled stations. The values of abundance and biomass recorded at the control stations in tidal channels of Kneiss islands (3187 ± 348 ind.m^−2^; 186 ± 22 g AFDW.m^−2^, respectively) appear to be very high compared with other Mediterranean ecosystems [46], showing that the tidal channels ecosystems form suitable and highly productive habitats for the macrobenthic fauna [16,47].

The seafloor morphologies in the tidal channels are of crucial importance for the functioning of marine ecosystems since they allow for the exchange of water, sediments, nutrients, biota, and pollutants with the open sea [16,48]. The low biodiversity of the macrozoobenthic fauna recorded at trawled stations reflects the strong impact of destructive fishing gear, potentially affecting entire habitats and leading to the damage and/or killing of benthic organisms. As a result, this reduces species taxonomic richness, abundance, and biomass as reported in several other international studies [49,50,51]. The notable increase in benthic community indices during the COVID-2019 pandemic lockdown period and after two days of deconfinement (5 June 2020) at trawled stations highlights the negative effects of illegal fishing gear on the subtidal benthic soft-bottom communities of the Kneiss Islands. However, in the absence of trawling activities, we observe an increase in the abundance of many species such as the tubicolous polychaetes *Euclymene oerstedii* and *Melinna palmata;* the infaunal polychaetes *Cirratulus cirratus* and *Scolemata impatiens;* the amphipod *Microdeutopus anomalus*; and the bivalves *Scrobicularia plana*, *Loripes orbiculatus*, and *Pinctada imbricata radiata.* Likewise, other numerous sedentary fauna such as the cnidaria, echinodermata, and tubicolous polychaeta species (e.g., *Sabellidae, Terebellidae,* and *Serpulidae*) appeared only at impacted stations during the lockdown period and after two days of deconfinement. In fact, the continuous fishing disturbance contributes to a decline in the resistance of the macrobenthic assemblages of the Kneiss Islands. The high resilience of these shallow benthic communities during the cessation of fishing activities is indicated by the reappearance of smaller and some sensitive species such as infaunal polychaetes (e.g., tubicolous species) and mobile crustaceans that are unable to withstand environmental changes due to their biological traits (life history, morphology, behaviour of each taxon, inter-specific interactions, and connections between species and their environment); this also proves that the stability of sediment and nutrients of the tidal channels in the absence of bottom trawling promotes the successful recruitment and colonization of several benthic species and increases the trophic availability, which also attracts other mobile organisms from neighbouring ecosystems (intertidal zones). The short-term increase in the number abundance, and biomass of many sensitive and small-sized benthic organisms during the COVID-19 lockdown period in the tidal channels of Kneiss could be explained by the fact that this period coincides with the recruitment period of the majority of the macrobenthic species that generally takes place during the spring and summer season [52]. Previous studies showed that opportunistic species, such as the polychaete families Spionidae and Cirratulidae, are characterized by high growth rates, a short life span, a low reproductive age, and a large reproductive output [53,54]; these are more resistant to change and impose their opportunistic strategy of life to overcome environmental changes caused by anthropogenic pressures including fishing disturbances [45,55].

Trawling stress leads to the selection of species that are adapted to living in soft-bottom habitats. Combined with a strong bottom trawling activity, this implies that the benthic community is able to recolonize this environment rapidly after the cessation of anthropogenic pressures [56]. In fact, mobile species (e.g., amphipods and small species) have the ability to tolerate environmental changes and are able to escape during fishing disturbances [57]. Moreover, the smaller and sensitive macrobenthic organisms are also vulnerable to trawling effects [45,58], often displaying relatively high growth capacity and turnover rates, resulting in shorter recovery times [42,59,60].

The reappearance of some macrobenthic species and the increases in abundance and biomass of macrofauna at the trawled stations during the COVID-19 lockdown suggest that one month without trawling activities is necessary for the restoration of biodiversity and for the recovery of the subtidal ecosystem of the Kneiss Islands. Many authors have shown the strong resilience capacity of benthic communities following the short impacts of fishing disturbances including bottom trawling effects and that the speed and nature of recovery can vary greatly, depending upon the type and duration of impact and upon the physical and life-history characteristics of the species making up the macrobenthic communities [6,60,61,62,63].

The depletion in diversity and biomass of macrobenthic communities (especially polychaetes; Cirratulidae, Nereididae, and Maldanidae) observed at trawled stations during the fishing period (almost −70% after a month of fishing) is due to direct and indirect mortality (destruction of tubes, exposure to predators, and habitat destruction) [63,64,65,66]. Generally, the diversity and abundance of tubicolous polychaetes decreases after any destructive fishing activity [20,37,65]. Environmental changes caused by destructive fishing gear lead to effects that modify the habitat structure, disrupt food web processes, and eliminate the more vulnerable organisms susceptible to environmental damage. This is because organisms are displaced by the fluidized sediments generated by the pressure wave in front of a moving trawl [67,68]. Usually, macrobenthos respond negatively to fishing pressures, since they are highly vulnerable and can be considerably reduced in diversity and abundance or even eliminated, being extremely fragile and particularly susceptible to damage [45,69]. The increase in macrobenthic fauna biomass at the trawled stations during the COVID-19 lockdown compared with the fishing periods confirms that chronic trawling has a strong negative impact on the biomass and the functional capacity of subtidal benthic macrofaunal communities. These results are in agreement with other previous studies reporting the negative effects of trawling activity on the diversity, size composition, and biomass of benthic communities [50,62]. Equally, biomass is an effective indicator of trawling disturbance. Recently, Sciberras et al. [50] demonstrated that the effects of trawling are most pronounced in long-lived benthic organisms, as these typically take longer to recover after a trawling event. In the tidal channels of the Kneiss Islands, the effects of trawling influence not only the sensitive taxa (infauna polychaetes) but also the larger macrobenthic communities such as the crustacean decapods *Carcinus* spp. and *Metapenaeus Monoceros* (Fabricius, 1798) and the molluscs *Pinctada imbricata radiata* (Leach, 1814) and *Cerastoderma glaucum* (Bruguière, 1789). Evenly, many experimental studies have revealed that large macrofauna are also disproportionately sensitive to trawling disturbance [70,71,72]. This vulnerability has been linked to a relationship between body size and several key life-history traits [73], whereby larger macrobenthic species tend to grow and reach maturity at a slower rate. Such species have comparatively lower mortality and population growth rates and are therefore more vulnerable to trawling-induced mortality. Repeated and intense trawling would typically result in shifts away from communities dominated by a high biomass of long-lived organisms towards those dominated by highly abundant small macrofauna including opportunistic species [42,71,74]. The frequently trawled tidal channels of the Kneiss islands show increased water turbidity caused by the scraping of the seabed during trawling and the injection of sediments into the water column [75] associated with a decrease in dissolved oxygen concentration [52]. The impact of oxygen deficiency results in various behavioural changes for macrofauna communities. Mobile organisms (i.e., amphipods and decapods) are able to move away from or avoid hypoxic locations, while the behavioural responses of sessile species (i.e., some molluscs, infauna, and tubicolous polychaetes) might express modifications of feeding and bioturbation rates [76]. However, complete faunal depletion is observed at low oxygen concentrations and under anoxic conditions in trawled zones [77]. Generally, various co-varying and interacting factors have been proposed to account for the environmental effects of bottom trawling. These may include the sensitivity of specific marine habitats [7,62], the impact of different gear types [1,78], the magnitude of background human and natural disturbances [52,79], and the capacity of benthic communities to adapt (resistance) to environmental disturbances [62,74]. Either alone or in combination, such factors could obscure the measurable effects of trawling on benthic habitats and organisms and may also explain why indicators sometimes display various different performances [12,80].

## 5. Conclusions and Future Perspectives

The present study shows the negative effects of bottom trawling on macrobenthic fauna in the tidal channels of the Kneiss Islands. This destructive fishing gear causes a notable reduction in the taxonomic richness, abundance, and biomass of the macrobenthic communities. It is well known that the macrofauna plays a crucial role in the food web either by feeding on detritus or as food for aquatic birds and economically important demersal fish [81]. Thus, it would be very important to ban this illegal destructive fishing activity in the Kneiss Islands, which represents a site of international interest in terms of marine biodiversity (Important Bird Area, SPAMI, and RAMSAR Site). This study shows that just one month of non-fishing is sufficient to allow for the recovery of benthic biodiversity. Finally, this exceptional lockdown period due to the COVID-19 pandemic points to the high resilience of the macrobenthic community after the cessation of trawling disturbances, which affects the interface between the sediment and the bottom layer of the water column and has widespread negative impacts on benthic communities and marine habitat. It could be a significant and important reason to prevent and prohibit the use of this illegal and destructive fishing gear to better protect the marine biodiversity of the tidal channels of Gulf of Gabès.

Several aims might be considered for future work. The first would be to produce an effective management plan integrating stakeholder communities to conserve the fisheries of the Kneiss Islands. Second, it would be interesting to complete an evaluation of the impacts of bottom trawling on macro- and megabenthic fauna in other tidal channels of the Gulf of Gabès (studied recently by Dauvin et al. [16]). Finally, it might be worthwhile carrying out seasonal and annual monitoring at selected stations along these tidal channels to follow the long-term response of macrofauna communities to illegal fishing pressures.

## Figures and Tables

**Table 1:** Mean environmental characteristics of the four sampling stations during the five campaigns of the March–June period.

Stations	Coordinates		Depth (m)	OM (%)	Q50 (mm)	Sandy (%)	Silt-Clay (%)	Sediment Type
	Latitude (N)	Longitude (E)						
**I1**	38.00974	32.614844	5.60	4.1	0.79	83.5 ± 3.62	16.0 ± 5.18	medium sand
**I2**	37.98597	32.615700	8.30	3.8	1.11	97.3 ± 4.10	2.6 ± 6.22	coarse sand
**C1**	37.96937	32.615471	7.40	3.6	1.21	96.8 ± 3.12	3.2 ± 4.60	coarse sand
**C2**	37.95854	32.614429	6.50	2.6	1.14	95.8 ± 2.64	3.4 ± 1.42	coarse sand

**Table 2:** Proportion of zoological groups (A: % total abundance; B: % total biomass), the mean taxa number, mean abundance, and mean biomass recorded at the two impacted (I) and two control stations (C) during the five sampling periods: The mean taxa number (number of taxa per 0.2 m² ± SD); the mean abundance (number of individuals per m² ± SD); the mean biomass (g AFDW per m^2^ ± SD); BT: bottom trawling and others groups (echinoderms, cnidarians, and tunicates).

	Presence of BT Activity				Absence of BT Activity				Deconfinement; Presence of BT Activity											
	Before Lockdown				Lockdown				After Two Days				After Two Weeks				After One Month			
	C		I		C		I		C		I		C		I		C		I	
	A	B	A	B	A	B	A	B	A	B	A	B	A	B	A	B	A	B	A	B
**Polychaetes**	47	35.1	27.0	14.4	42	34.3	37	24.9	36	35.4	30	21.8	43	31.7	26	16.2	49	31.3	19	13.6
**Crustaceans**	27	34.3	44	51.3	33	35.1	35	42.8	38	34.5	39	42.4	31	36.0	42	50.4	26	38.3	42	54.2
**Molluscs**	22	27.2	27	32.2	21	28.1	25	28.8	24	28.3	30	34.6	23	30.3	32	33.4	22	27.2	39	32.2
**Others groups**	4	3.4	2	2.1	4	2.4	3	3.5	2	1.8	1	1.2	3	2.0	0	0	3	3.2	0	0
**Mean taxa number**	35.0 ± 3.0		11.0 ± 2.1		41.5 ± 3.6		33.0 ± 4.2		36.0 ± 2.4		25.5 ± 2.6		36.5 ± 3.2		17.0 ± 1.8		34.5 ± 3.4		9.5 ± 2.2	
**Mean abundance**	2762 ± 202		1256 ± 112		3187 ± 348		2807 ± 211		2856 ± 262		1923 ± 104		2821 ± 301		1112 ± 96		2634 ± 182		958 ± 62	
**Mean biomass**	113 ± 20		64 ± 9		186 ± 22		124 ± 12		194 ± 24		102 ± 13		198 ± 14		68 ± 8		211 ± 14		49 ± 6	

**Table 3:** Results of the ANOVAs with the three factors: time (5 levels; df = 4), trawling (2 levels; df = 1), and site (4 levels; df = 3) for the five biological variables (*: significant variation).

	Factor	*F*	*p*
	Time	3.9	<0.01 *
Richness species	Site	1.14	<0.01 *
	Trawling	0.23	0.01 *
	Time	2.9	<0.01 *
Abundance	Site	1.21	<0.01 *
	Trawling	0.84	<0.01 *
	Time	21.4	0.01 *
Biomass	Site	0.86	<0.01 *
	Trawling	12.2	0.01 *
	Time	2.11	<0.01 *
H’	Site	2.18	<0.01 *
	Trawling	1.14	0.01 *
	Time	2.41	0.01 *
J’	Site	3.11	<0.01 *
	Trawling	1.04	0.01 *

**Table 4:** Groups formed by n-MDS ordination, with indication of similarities between each group (%) and the most representative species (% of contribution for the similarity within the group) contributing to the similarity within the group, determined with SIMPER analysis.

	GI	GII	GIII
**Similarity (%)**	**64.54**	**49.43**	**27.82**
**Main species**	*Cirratulus cirratus*—52.3	*Cirratulus cirratus*—48.6	*Microdeutopus gryllotalpa*—51.6
	*Euclymene oerstedii*—46.0	*Hilbigneris gracilis*—44.9	*Cymadusa filosa*—48.2
	*Hediste diversicolor*—38.1	*Perinereis cultrifera*—42.0	*Dexamine spinosa*—41.0
	*Amphitritides gracilis*—34.2	*Cerithium scabridum*—37.0	*Gibbula ardens*—32.2
	*Leucothoe incisa*—28.2	*Euclymene oerstedii*—26.7	*Tellina tenius*—28.1
	*Eulymene lumbricoides*—26.6	*Scrobicularia plana*—21.0	*Tritia cuvierii*—21.4
	*Monocorophium acherusicum*—22.7	*Melita palmata*—18.2	*Gammarus insensibilis*—20.8
	*Microdeutopus anomalus*—18.6	*Monocorophium insidiosum*—16.1	*Euclymene oerstedii*—19.4
	*Scrobicularia plana*—11.3	*Marphysa sanguinea*—10.2	*Loripes orbiculatus*—17.3
	*Melinna palmata*—7.0	*Tricolia speciosa*—8.6	*Calliostoma zizyphinum*—15.2
	*Heteromastus filiformis*—4.2	*Dexamine spinosa*—3.2	*Pinctada (imbricata) radiata*—7.1

## Data Availability

All data used are in the manuscript and are available from the corresponding author.
